# Modified Carbon Fiber Paper-Based Electrodes Wrapped by Conducting Polymers with Enhanced Electrochemical Performance for Supercapacitors

**DOI:** 10.3390/polym10101072

**Published:** 2018-09-27

**Authors:** Sicong Tan, Jiajia Li, Lijie Zhou, Peng Chen, Jiangtao Shi, Zhaoyang Xu

**Affiliations:** College of Materials Science and Engineering, Nanjing Forestry University, Nanjing 210037, China; 17768109951@163.com (S.T.); jiajiaLinjfu@163.com (J.L.); zhoulijie6688@163.com (L.Z.); 15605170735@163.com (P.C.); shijt@njfu.edu.cn (J.S.)

**Keywords:** carbon fiber paper, cellulose, carbon nanotubes, polyaniline, supercapacitor

## Abstract

An easy approach to fabricating carbon fiber paper (CFP) based electrodes has been developed. This method can be mainly divided into two steps, for which the mixture of cellulose nanofibers (CNFs) and carbon nanotubes (CNTs) was first deposited on the surface of carbon fiber paper through a vacuum filtration device followed by immersing the hybrid paper into concentrated aniline solution to polymerize polyaniline (PANI). Compared to carbon fiber paper, the acid-treated carbon fiber paper (A-CFP)-based electrode provides more active sites, which are beneficial for the polymerization of polyaniline. The mixture of CNFs and CNTs could coat on the A-CFP by vacuum-filtration due to the high hydrophilicity of A-CFP improved by acid-treatment. PANI with different polymerization time was in-situ synthesized on the surface of the hybrid paper to form a three-dimensional cross-linked structure that greatly enhanced the electrochemical performance of the electrode by improving high capacitance, high rate-capability, and long cycle-life. Moreover, the assembled symmetrical supercapacitor showed a high area capacitance of 626 mF·cm^−2^ and an energy density of 87 µWh·cm^−2^. This facile, easy performed, and low-cost strategy may provide a feasible method for the production of supercapacitor electrodes.

## 1. Introduction

To overcome the severe issue of the rapid consumption of oil resources and global warming problems, much effort has been paid to the development of energy storage devices. Supercapacitors, as an important class of energy-storing devices, have attracted great interest in recent years due to their numerous superior properties such as large capacitance, high power-density, fast charging/discharging rate, and long cycle-life [1,2,3,4]. Chiefly, supercapacitors can be divided into two types: Electrical double layer capacitors (EDLC) store energy through fast ion adsorption, while pseudocapacitors work via reversible surface Faradic reactions [5]. Compared with EDLC, pseudocapacitors generally possess higher capacitance and power density considering their electrochemical nature and thus catch more attention [6].

Mainly, pseudocapacitor materials can be categorized into two types: (i) conducting polymers and (ii) transition metal oxides. Through transition metal oxides, RuO_2_, MnO_2_, and Co_3_O_4_ have attracted most attention due to their high theoretical capacitance, and much pioneer work has been done. For instance, Manjusha V. Shelke and his co-workers have successfully synthesized the RuO_2_/CNOs nanocomposite using a sol-gel method, and a high capacitance of 570 F·g^−1^ for the composite electrode has been achieved in 0.5 M H_2_SO_4_ aqueous solution [7]. Fabrication of RGO/MnO_2_ paper has also been made using a free template and simple synthesis process, and a high area capacitance of 897 mF·cm^−2^ has been acquired by Pooi See Lee and his group [8]. For supercapacitors electrodes made by mesoporous Co_3_O_4_ nanosheet decorated with hydrous RuO_2_ nanoparticle, an enhanced rate performance of 78% is achieved at current density ranging from 1 to 40 A·g^−1^ [9]. However, transition metal oxides suffer from poor power density and cycling [10].

Considering conducting polymers, polyaniline has stood out due to its unique properties including high conductivity, high capacitance, easy synthesis, low cost, and environmental friendliness [11]. Nevertheless, carbon nanotubes are often used as enhancers to improve the electrochemical performance of PANI due the low stability of PANI during charging/discharging process [12]. For example, Wang has designed supercapacitor electrodes based on PANI and carbon black composites through a one-step potentialdynamic co-deposition process, and a high capacitance of 458 F·g^−1^ has been achieved at 2 mV·s^−1^ [13]. Compared to carbon black, CNTs have been used extensively to improve the electrochemical performance of PANI due to its high conductivity, high aspect ratio, high mechanical strength, and chemical stability [14,15]. Significant efforts have been devoted to the fabrication of CNTs/PANI electrodes [16,17]. Moreover, CNTs have been demonstrated to show superior properties when used as electrode materials, since they lower the resistance and increase the energy efficiency of electrodes [18].

Both PANI and CNTs suffer from low hydrophobicity, which is not beneficial for contact between electrodes and electrolytes [19,20]. Cellulose, as the most abundant and sustainable biopolymer on earth, has attracted much attention due to its several remarkable properties such as high aspect ratio, good mechanical properties, low cost, and superior hydrophilics, and has been applied to many fields including the textile industry, the biomedical field, and typically, wearable and flexible electronic devices [21,22,23,24,25]. The mesoporous structure of CNFs can provide diffusion channels for electrolytes, and its excellent hydrophilic property may help improve the hydrophobicity of CNTs and PANI.

In this work, we report a simple and low-cost “vacuum-filtering and in situ growth” method to fabricate carbon fiber paper based electrodes. CFP was first activated by H_2_SO_4_/HNO_3_ mixed acid to improve its poor hydrophobicity and low surface area. Then, an extremely thin layer of CNFs/CNTs hybrid mixture was deposited on the surface of CFP, and the hybrid electrode was later immersed into concentrated aniline solution. After several hours of polymerization, the supercapacitor electrodes could be achieved. The thin layer of CNFs/CNTs mixture not only provides diffusion channels for the electrolyte ions but also offers extra conducting channels that may enhance the electrochemical performance of the electrode. The resulting A-CFP/CNFs-CNTs/PANI electrode exhibits huge area capacitance and excellent cycle ability. FT-IR spectroscopy, X-ray diffraction, XPS, Raman, and BET measurement were used to characterize chemical properties of the A-CFP/CNFs-CNTs/PANI electrode, while cyclic voltammetry (CV), galvanostatic charge-discharge (G-CD), and electrochemical impedance spectroscopy (EIS) were used to evaluate its electrochemical quality.

## 2. Experimental

### 2.1. Materials

The raw CFP for the preparation of functionalized carbon fiber paper (thickness 0.02 mm, density 0.78 g·cm^−3^, resistivity 2.5 mΩ·cm^−2^) was bought from Shanghai Hesen Electric. Co. Ltd., Shanghai, China. Raw bamboo powder provided by Hangzhou gaoke composite co. LTD, Zhejiang, China was obtained for the production of cellulose nanofibers. Multiwalled carbon nanotubes were achieved from Shenzhen Nanotech Port Co., Ltd., Shenzhen, China. Pure aniline monomers and ammonium persulfate were obtained from Shanghai Ling Feng Chemical Reagent Co., Ltd., Shanghai, China. All the reagents were in analytical grade and used without purification.

### 2.2. Preparation of Activated Carbon Fiber Paper

The activation process of CFP was performed according to the literature [26]. Just in brief, the raw CFP was cut into small pieces with a geometric area of 4.5 × 4.5 cm^2^. After washed several times with deionized water and ethanol, the CFP was dried at 60 °C for 12 h. The pre-treated CFP was then immersed into a mixed acid containing both sulfuric acid and nitric acid (volume ration 3:1) at 60 °C for 1 h. The acid-treated CFP was denoted as A-CFP.

### 2.3. Preparation of Cellulose Nanofibers

The synthesis process was performed according to the literature and adapted with small modifications [27]. Just in brief, 10 g of bamboo powder was added into 500 mL water containing 10 g KOH (98%) and intensively stirred at 75 °C for 1 h for five times to remove lignin from bamboo powder. Then, a certain amount of potassium hydroxide was added into the solution and mechanical stirred at 90 °C for 2 h to remove hemi-cellulose and other impurities. Further, the sample was treated with acidified sodium chlorite (5 g, 98%) solution at 75 °C for 1 h and potassium hydroxide at 90 °C for 2 h to improve its purity. Lastly, 12 mL hydrochloric acid (2 mL, 98%) was added into the solution and kept stirring at 80 °C for 2 h. After chemical treatment, the samples were mechanically polished to get a CNFs slurry. Deionized water was used to remove excess reagent at every step to keep pH of the sample neutral.

### 2.4. Preparation of A-CFP Based Electrodes

**Figure 1:**
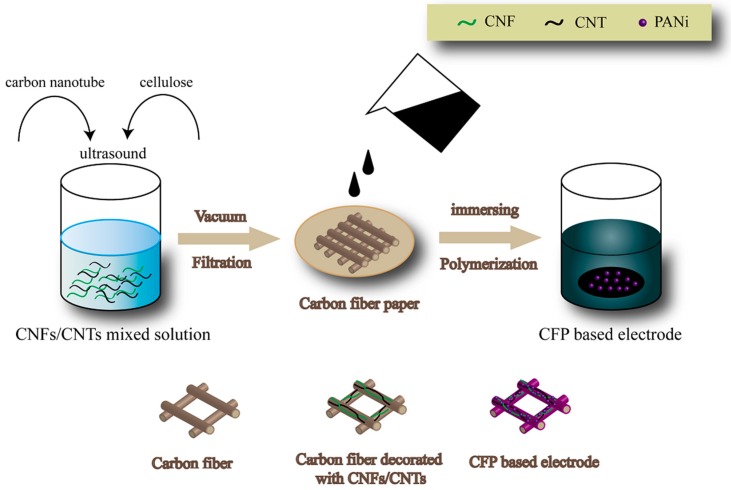
Experimental process of the samples.

The experimental process is shown in Figure 1. All the chemicals were of analytical grade and used without further purification. In a typical process, 10 mg CNFs and 10 mg CNTs (mass ration = 1:1) were added into 100 mL deionized water followed by adding 40 mg sodium dodecyl benzene sulfonate (SDBS) as dispersant. The mixed solution was ultrasounded for 30 min to achieve a homogeneous solution and was then coated on the surface of A-CFP through a vacuum filtration device. CNFs/CNTs deposited on A-CFP with different weight was denoted as CCM_p-5_, CCM_p-10_, and CCM_p-15_, respectively. Then, 5 mmol aniline monomers were dissolved in 100 mL 1 M HCl and magnetically stirred for several seconds. A piece of CCM_p-10_ was immersed into the solution as mentioned above to adsorb aniline monomers for an hour followed by adding 5 mmol ammonium persulfate for the polymerization of PANI. The polymerization was carried out for 2, 4, 6, 8, and 24 h, and the obtained samples were denoted as CCM_s-2_, CCM_s-4_, CCM_s-6_, CCM_s-8_, and CCM_s-24_ respectively. The whole hybrid paper of CCM_s-2_, CCM_s-4_, CCM_s-6_, CCM_s-8_, and CCM_s-24_ weighted about 14.5, 15.6, 16.5, 18, and 21.2 mg·cm^−2^ (for CCM_s-x_, x means the polymerization time of PANI). PANI was also polymerized directly on the surface of A-CFP without CNFs/CNTs for the purpose of comparison and was denoted as C_p-6_.

### 2.5. Assemble of the Symmetric Supercapacitors (ASSC)

5 mL H_2_SO_4_ and 5 g polyvinyl alcohol was added into 10 mL deionized water and mechanically stirred for 2 h at 85 °C to achieve clear gel electrolyte. The CFP based electrodes were cut into rectangular shapes (2 × 1 cm^2^) and then immersed into gel for 30 min. The two electrodes were air dried at room temperature for about 4 h to evaporate the excess water and compressed together at 0.2 MPa for 10 min to form the symmetric supercapacitor.

### 2.6. Chemical Characterization and Electrochemical Measurements

Functional groups of synthesized electrodes were detected using a FTIR spectrometer with an attenuated total reflectance (ATR) device in the wave number range from 4000 to 500 cm^−1^ (Nicolet iS10, Thermo Electron Corp., Waltham, MA, USA). XRD patterns of the samples were conducted using an X-ray diffractometer with Cu Ka radiation (40 kV and 30 mA) at a scanning rate of 5°/min (Ultima IV, Rigaku, Tokyo, Japan). Photoelectron spectroscopy (XPS) and Raman spectroscopy analyses were also performed to understand the surface distribution of functional groups and the molecular structure. A field emission scanning electron microscopy (FE-SEM, S-4800, HITACHI, Tokyo, Japan) was conducted at 5.0 kV to characterize the morphology and structure of the samples. Brunauer-Emmett-Teller (BET) measurement was conducted using an ASAP 2020 V3.00 H surface area analyzer to determine the N_2_ adsorption/desorption.

The electrochemical performances of the samples were characterized using a three-electrode system. Mainly, CCM_s-x_ were cut into 2 × 1 cm^2^ and used directly as working electrodes without any conductive binders. Aqueous solution of 1 M H_2_SO_4_ was used as electrolyte. Cyclic voltammetry (CV), galvanostatic charge−discharge (GCD), and EIS were tested using a CHI660E electrochemical workstation (from Chenhua, Shanghai, China) to determine the electrochemical performances of the samples. The electrochemical performances of the ASSC was tested in a two-electrode system using Na_2_SO_4_ as electrolyte.

The specific capacitance, energy densities, and power densities of the samples were calculated using the following equations:

(1) $$C_{A}=Q/A\times \Delta V=\frac{\int IdV}{vs\Delta V}$$

(2) $$C_{A}=Q/A\times \Delta V=\frac{I\Delta t}{S\Delta V}$$

(3) $$E=\frac{1}{2}C_{A}{\left(\Delta V\right)}^{2}$$

(4) $$P=\frac{E}{\Delta t}$$

in which *C_A_* represents the specific area capacitance of the electrodes, *P* represents power density, *E* means energy density, ∫IdV means the integral area of *CV* curves calculated by origin software, *s* is the geometric area of the electrodes, *v* is the potential sweep rate, and ∆*V* is a total potential deviation of the voltage window (including IR drop). *I* is the constant discharge current, while ∆*t* means the discharge time.

## 3. Results and Discussion

### 3.1. SEM Images

**Figure 2:**
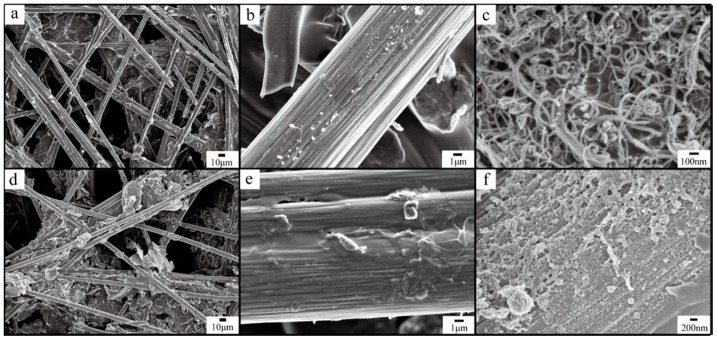
SEM images of low magnification of raw carbon fiber papers (**a**), high magnification of raw carbon fiber papers (**b**), CNFs/CNTs mixture deposited on the surface of A-CFP (CCM_p-10_) (**c**), low magnification of activated carbon fiber papers (**d**), high magnification of activated carbon fiber papers (**e**), and the opposite side of CCM_p-10_ (**f**).

The morphology of the CFP (Figure 2a,b), A-CFP (Figure 2d,e), and CCM_p-10_ (Figure 2c,f) are shown in Figure 2 with different magnifications. The diameter of carbon fibers of both CFP and A-CFP are 7 micrometers approximately and much polytetrafluoroethylene (PTFE) can be observed filling in the vacancy among the carbon fibers, which is a necessary binder material for the production of CFP. Oxygen-containing groups were functionalized on the surface of the CFP due to the following reaction (5):

(5) $$\mathrm{CFP}+H_{2}{\mathrm{SO}}_{4}/{\mathrm{HNO}}_{3}\to \mathrm{CFP}-\mathrm{OH}/\mathrm{CFP}-\mathrm{COOH}$$

Further, the morphology of A-CFP shown in Figure 2d remained unchanged but became rougher, which can be seen in Figure 2e. For CCM_p-10_ (Figure 2c), both CNFs and CNTs with a diameter range between 10 and 50 nm can be identified fully covered on the surface of A-CFP. Moreover, the 3D porous network structure composed by crossed entangled CNFs and CNTs provided more active sites for the adsorption of PANI. Figure 2f confirmed the attachment of CNFs on the surface of a single carbon fiber, which demonstrated that the mixture of CNFs/CNTs penetrated into the deep inner space of carbon fiber paper. Differed from A-CFP, the deposited CNFs/CNTs mixture with porous 3D network provided large surface area (97.8 m^2^·g^−1^) for the polymerization of PANI. Moreover, the good hydrophilicity of CNFs may improve the contact between active materials and electrolytes and reduce the diffusion distance of electrolyte ions during the charge/discharge process.

**Figure 3:**
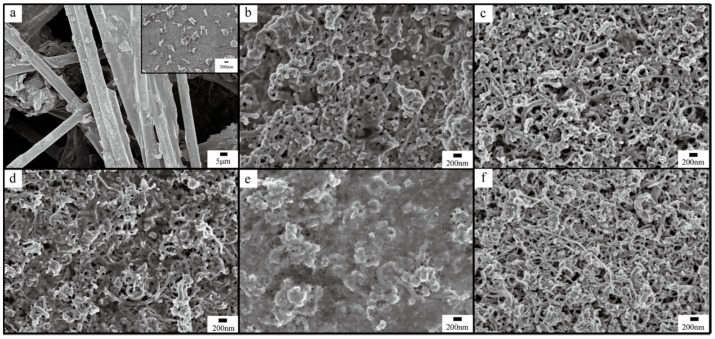
SEM images of PANI directly polymerized on the surface of A-CFP (C_p-6_) (**a**), CCM_s-2_ (**b**), CCM_s-4_ (**c**), CCM_s-6_ (**d**), CCM_s-8_ (**e**), and CCM_s-24_ (**f**).

Morphology of PANI directly coated on A-CFP is shown in Figure 3a. PANI with a diameter of approximately 600 nm was partially deposited on the surface of A-CFP. The inset of Figure 3a shows the enlarged photograph of C_p-6_. Morphology of PANI with different polymerization times deposited on the surface of CCM_p-10_ are shown in Figure 3b–f. As shown in Figure 3b, PANI layers were first coated onto the surface of CNFs and CNTs to form a core-shell nanostructure via in situ polymerization. The diameter of PANI coated on the CNFs and CNTs ranged between 100 and 200 nm. The construction of this interconnected three-dimensional conducting network made electrons transfer not only via junctions of CNTs but also through PANI and thus a minimized junction contact resistance could be achieved. Furthermore, this unique structure could act as a buffering layer to prevent the severe agglomeration of PANI, and the matrix of CNFs/CNTs could reduce the swelling and shrinking of PANI during the charging/discharging process. In Figure 3c, the structure of CCM_s-4_ became more compact and porous, which indicated the higher conversion degree of aniline monomer to PANI. For CCM_s-6_, the PANI layer became denser and less porous, which can be observed from Figure 3d. For longer polymerization time of 8 h, the fiber-like PANI totally disappeared, while large-scale of agglomeration could be seen, which have been due to the strong hydrogen bonding between sequentially formed PANI layers. For CCM_s-24_, PANI was densely packed, and an extra criss-cross structure could be observed.

### 3.2. FTIR Analysis

**Figure 4:**
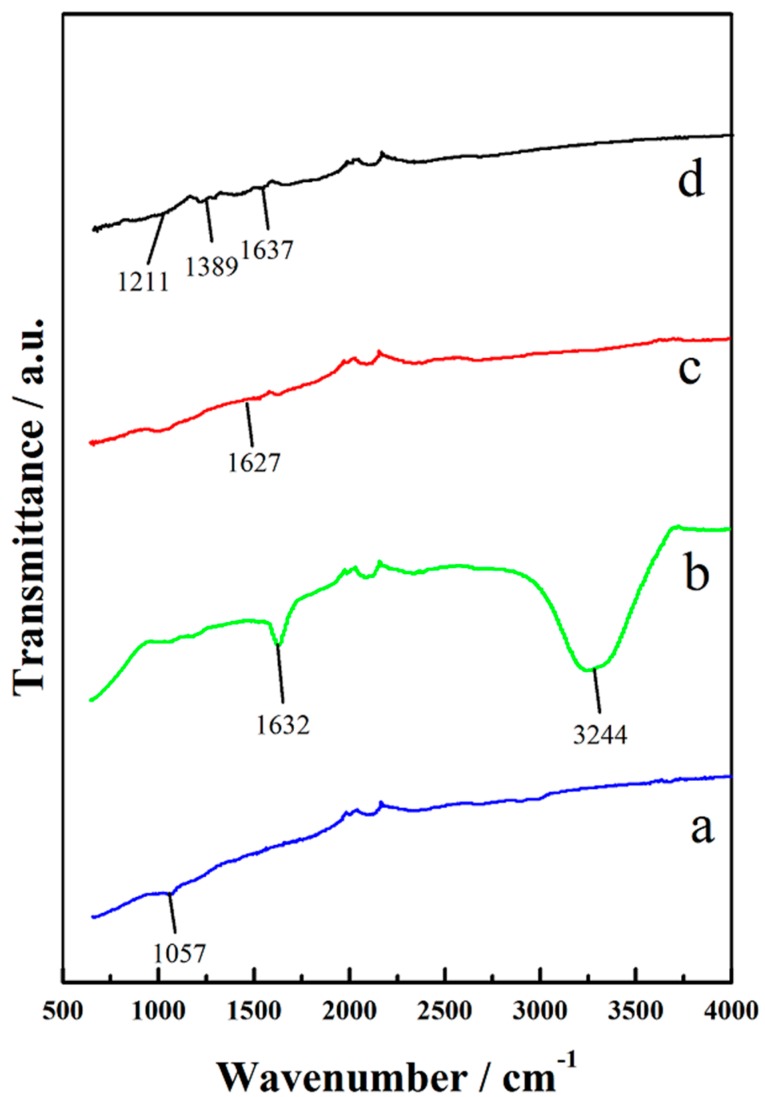
FTIR spectra of the raw CFP (**a**), A-CFP (**b**), CCM_p-10_ (**c**), and CCM_s-6_ (**d**).

The typical FTIR spectra of CFP is shown in Figure 4a, in which peaks centered at 1057 cm^−1^ represented the vibrational mode of C–O groups. In Figure 4b, the appearance of strong peak located at 1632 cm^−1^ was caused by the carbonyl group (C=O), and the broad peak from 2900 to 3700 cm^−1^ was attributed to N–H bonds, which confirmed the successfully oxidation of raw CFP with the mixed solution of H_2_SO_4_ and HNO_3_, and this was further confirmed by XPS [26,28]. Figure 4c exhibited the FTIR spectra of CCM_p-10_; the oxidation peak at 1627 cm^−1^ was obviously weakened, and the peak at 3244 cm^−1^ disappeared totally; thus, a relatively smooth curve can be observed, which may due to the coverage and symmetric structure of CNTs [29]. Characteristic peaks of PANI centered at 827, 1194, 1282, and 1520 cm^−1^ can be seen in Figure 4d, and they were caused by N–H vibration of the secondary amine, the bending of C–H group, C–N stretching of secondary aromatic amines, and the stretching of N=Q=N groups, respectively [30].

### 3.3. XRD Analysis

**Figure 5:**
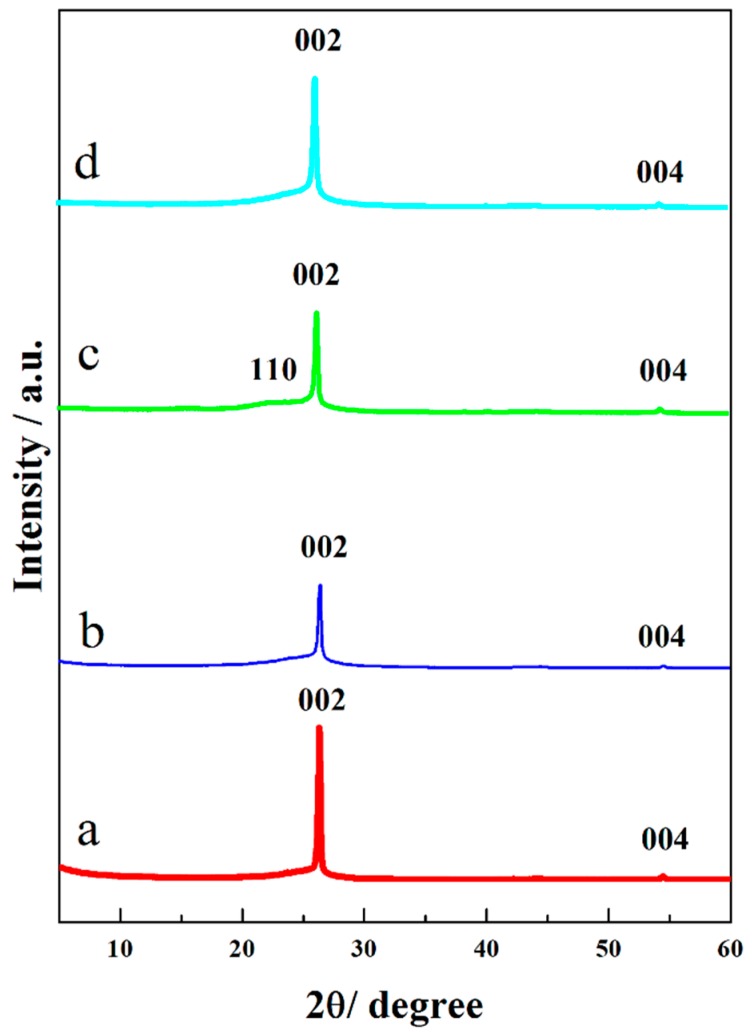
XRD patterns of raw CFP (**a**), A-CFP (**b**), CCM_p-10_ (**c**), and CCM_s-6_ (**d**).

XRD patterns were used to characterize the possible phase of the products. For CFP and A-CFP, peaks centered at about 26.3° and 54.5° can be indexed to a hexagonal graphite (JCPDF # 41-1487), and no other peaks were detected suggesting good purity of the samples. Compared to CFP, the peak intensity of A-CFP was much lower, which indicated the degradation of high crystalline-structure of graphite [31]. In Figure 5c, typical peaks of CFP were still detected, but the intensity became lower, which may have been due to the coverage of CNFs/CNTs mixture, and the new emerged peak located at about 22.5°, which was attributed to the typical cellulose I structure [32]. However, peaks of CNFs completely disappeared, and peaks of PANI did not show up at all (Figure 5d). With comparison to CCM_p-10_, XRD pattern of CCM_s-6_ showed typical peaks of CFP and A-CFP with increased intensity, which may have been caused by the different scales among the components.

### 3.4. Raman Analysis

**Figure 6:**
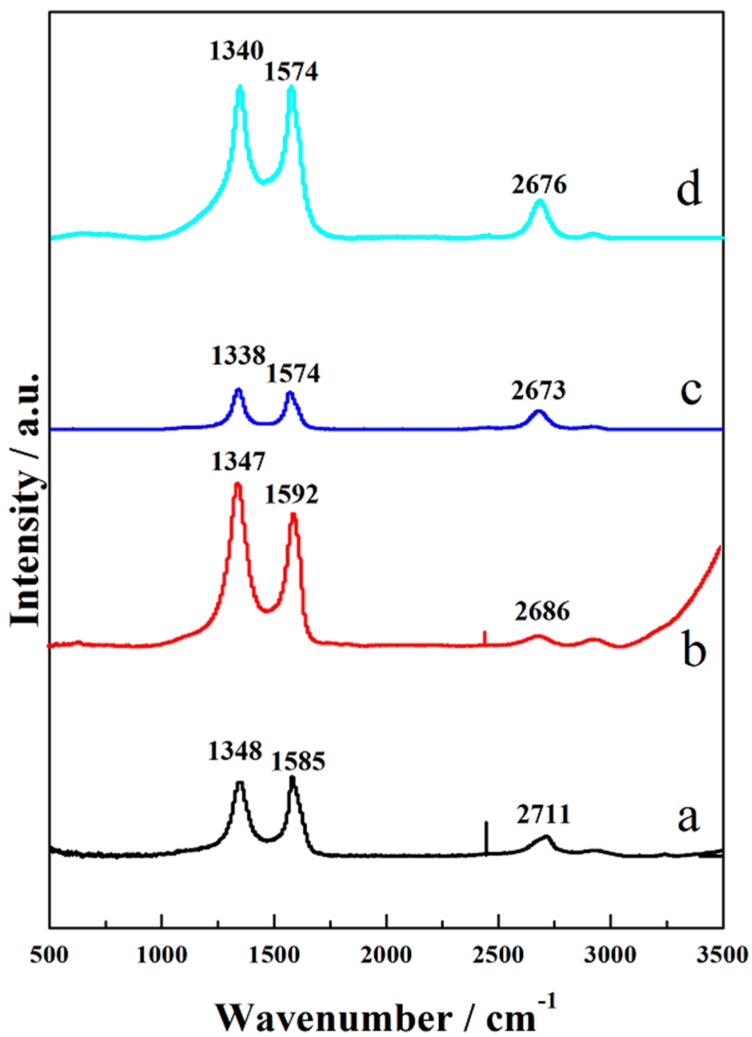
Raman spectra of the raw CFP (**a**), A-CFP (**b**), CCM_p-10_ (**c**), and CCM_s-6_ (**d**).

Raman spectra was captured to further characterize the surface structure of the samples. In Figure 6a, typical peaks centered at 1348, 1585, and 2711 cm^−1^ corresponded to the D, G, and 2D bands of carbon materials. Moreover, the D-band was related to the presence of disorder in sp^2^-hybridized carbon of carbon fibers, while G-band was caused by the hexagonal ring of the graphitic sp^2^ carbon fibers. For A-CFP in Figure 6b, the ratio of *I_D_*/*I_G_* and *I*_2*D*_/*I_G_* increased from 0.91 to 1.2 and decreased from 0.25 to 0.05, respectively, which implied that more defects were introduced into the graphite planes [30]. In Figure 6c, peaks centered at 1338, 1574, and 2673 were all in accordance with Raman spectra of CNTs and were caused by the disorder-induced D-band, the derived graphite tangential G-band, and the second-order harmonic G′-band, respectively [33]. Considering Raman spectra of CCM_s-6_ in Figure 6d, all the peaks almost remained at their location in Figure 6c, but the intensity became much higher, which may confirm the good combination among the components.

### 3.5. XPS Analysis

**Figure 7:**
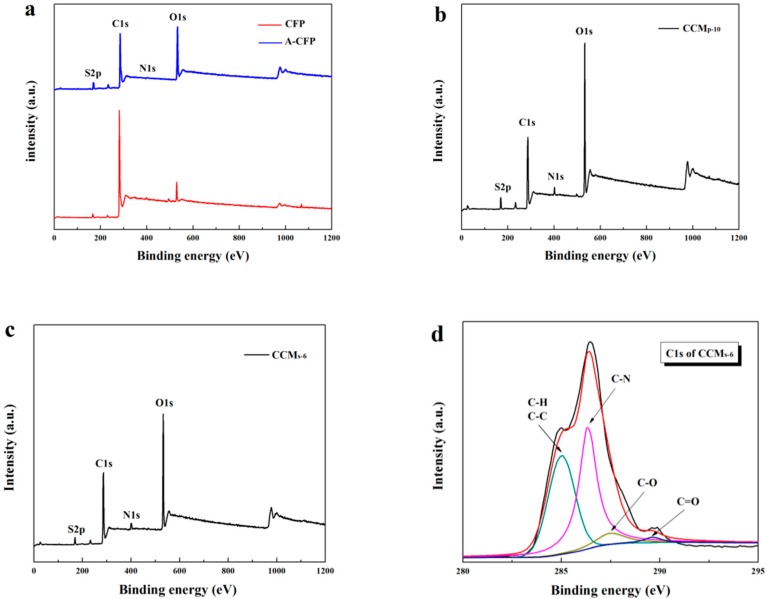
X-ray photoelectron spectra of the raw CFP and A-CFP (**a**), CCM_p-10_ (**b**), CCM_s-6_ (**c**), and narrow scan C1s of the CCM_s-6_ (**d**).

X-ray photoelectron spectra was used extensively to further characterize the surface functional groups of the samples. Figure 7a showed the wide scan XPS spectra of the raw CFP and A-CFP. Typically, after chemical treatment by mixed H_2_SO_4_/HNO_3_, the intensity of C1s of the raw CFP decreased, while the intensity of O1s increased, which indicated that more oxygen-containing groups were introduced into A-CFP. Moreover, the arising peak of N1s in the A-CFP suggested that HNO_3_ and H_2_SO_4_ can provide nitrogen and sulfer containing groups on the surface of CFP [34]. XPS spectra of both CCM_p-10_ and CCM_s-6_ are shown in Figure 7b,c. The intensity decrease of O1s in A-CFP may demonstrate that sufficient PANI was deposited on the surface of CNFs/CNTs. For narrow C1s XPS spectrum of CCM_s-6_, the binding energy peak centered at 284.5 eV can be mainly attributed to the in-plane C–C bending or the C–H bonds. The wide peak centered at 286.3 eV can be assigned to the C–N bond [35]. Peaks centered at about 287.5 and 289.6 eV derived from the carboxyl and hydroxyl groups bonded with carbon fibers [36].

### 3.6. BET Analysis

**Figure 8:**
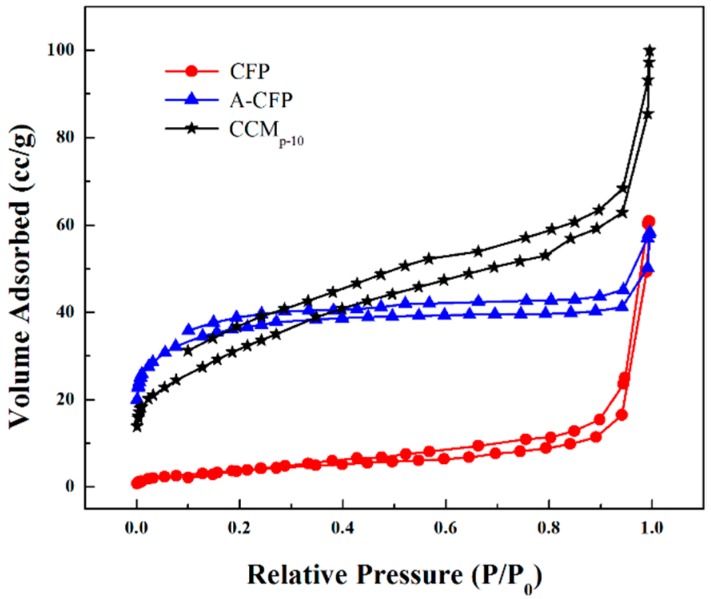
BET Nitrogen adsorption/desorption isotherm of the raw CFP, the A-CFP, and the CCM_p-10_.

A nitrogen gas adsorption/desorption technique was used to test the specific surface area of the obtained samples. As shown in Figure 8, the adsorption isotherm of all the materials were in type IV according to IUPAC. The specific surface area of the activated carbon fiber paper was measured to be 133.3 m^2^·g^−1^, which was approximately 8.5-fold higher than the raw carbon fiber (15.9 m^2^·g^−1^). This demonstrated that the acid treatment of mixed H_2_SO_4_ and HNO_3_ indeed increased the surface area of CFP. For CCM_p-10_, the surface area decreased little (116.2 m^2^·g^−1^) compared to A-CFP. Although the deposited mixture of CNFs/CNTs did not increase the specific of the material, it provided more active sides for the growth of PANI. This result was consistent with the SEM image and was beneficial for energy-storage devices.

### 3.7. Electrochemical Characterization

**Figure 9:**
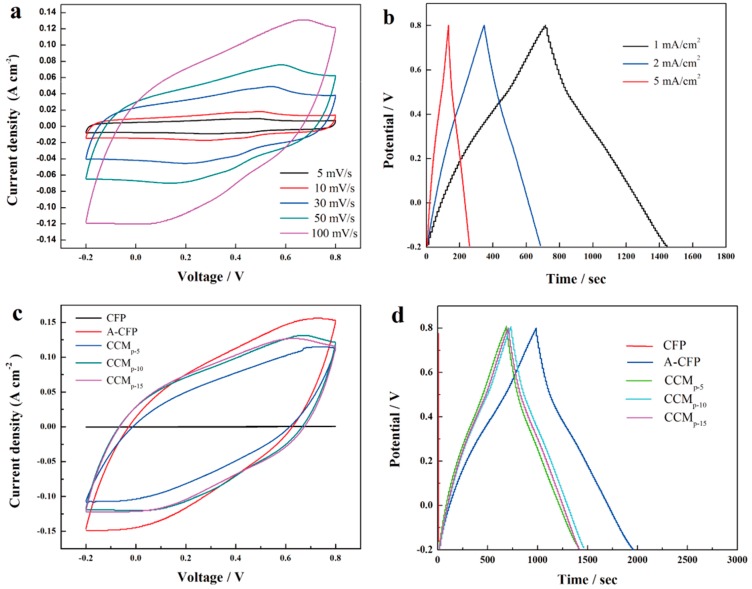
CV loops of CCM_p-10_ (**a**); GCD tests of CCM_p-10_ (**b**); comparison of CV loops of the raw CFP, A-CFP, CCM_p-5_, CCM_p-10_, and CCM_p-15_ (**c**); and comparison of GCD tests of the raw CFP, A-CFP, CCM_p-5_, CCM_p-10_, and CCM_p-15_ (**d**).

Electrochemical performances of the samples were evaluated using an electrochemical workstation (CHI660E) in 1 M H_2_SO_4_ electrolyte, with a Pt electrode as the counter electrode and a saturated calomel electrode (SCE) as the reference electrode. The total mass of CNFs/CNTs mixture deposited on the surface of A-CFP was fixed to be 20 mg (weight ratio 1:1). As depicted in Figure 9a, CV loops of CCM_p-10_ at different scan rates all showed a nearly symmetrical rectangle shape, which indicted an ideal capacity property. The capacitance of CCM_p-10_ was determined to be approximately 1300, 1235, 1057, 927, and 697 mF·cm^−2^ at 5, 10, 30, 50, and 100 mV·s^−1^, respectively. For comparison, CV curves of CFP, A-CFP, CCM_p-5_, CCM_p-10_, and CCM_p-15_ performed at 100 mV·s^−1^ are shown in Figure 9c. The pure CFP showed a small capacitance of 2.28 mF·cm^−2^, while A-CFP possessed a much higher capacitance of 740 mF·cm^−2^, which was almost 325 times larger than the former. This can be mainly attributed to the increase of surface area that benefits for the EDL capacity. The obvious redox pair derived from PANI appeared in the CV curve and analysis of XPS confirmed that the extra capacitance was provided by pseudocapacitive contribution derived from the oxygen-containing functional groups of A-CFP [33]. The detailed mechanisms are explained by the following reactions (6)–(8) [26]:

CFP–COOH ⇄ CFP–COO + H^+^ + e^−^ (6)

CFP–OH ⇄ CFP=O + H^+^ + e^−^ (7)

CFP=O + e^−^ ⇄ CFP–O^−^ (8)

When it comes to CCM_p-5_, CCM_p-10_, and CCM_p-15_, smaller CV areas can be observed, which may be due to the insulativity of CNF, but a high capacitance of 507, 697, and 703 mF·cm^−2^ can still be achieved. GCD tests were further used to characterize the charging/discharging performance of the samples. As it is clearly seen in Figure 9b, all the GCD curves are closely linear, and no obvious voltage drop can be observed that further identifies the good electrochemical performance of CCM_p-10_. A high specific-area capacitance of 730, 682, and 630 mF·cm^−2^ was achieved, respectively, as the discharge currents verified from 1 to 5 mA·cm^−2^. These results are comparable with those obtained from CV loops, and about 86.3% of the initial capacity was retained when it was charged to 5 mA·cm^−2^, which indicated the good rate capability of the free-standing electrode. Comparison of GCD curves of CFP, A-CFP, CCM_p-5_, CCM_p-10_, and CCM_p-15_ are exhibited in Figure 9d. A high capacitance of 976 mF·cm^−2^ at 1 mA·cm^−2^ for A-CFP was achieved, which was nearly 1621 times higher than the raw CFP. A relatively similar value of 721, 730, and 707 mF·cm^−2^ was obtained for CCM_p-5_, CCM_p-10_, and CCM_p-15_. Although the existence of CNFs/CNTs lowers the capacitance of the A-CFP, which may due to the high resistance of CNFs, but the high hydrophilic property of CNFs may enhance the rate capability of A-CFP [37]. Typically, with the increasing of discharge current from 1 to 5 mA·cm^−2^, an increased rate capability of 80.0%, 86.3%, and 87.0% was achieved compared to A-CFP (83%). Considering that further increment of the weight of CNFs/CNTs mixture may lower the capacitance overmuch, we choose CCM_p-10_ as the substrate to further polymerize PANI, and the deposited CNFs/CNTs mixture is expected to improve the rate capability and cycling performance of PANI.

**Figure 10:**
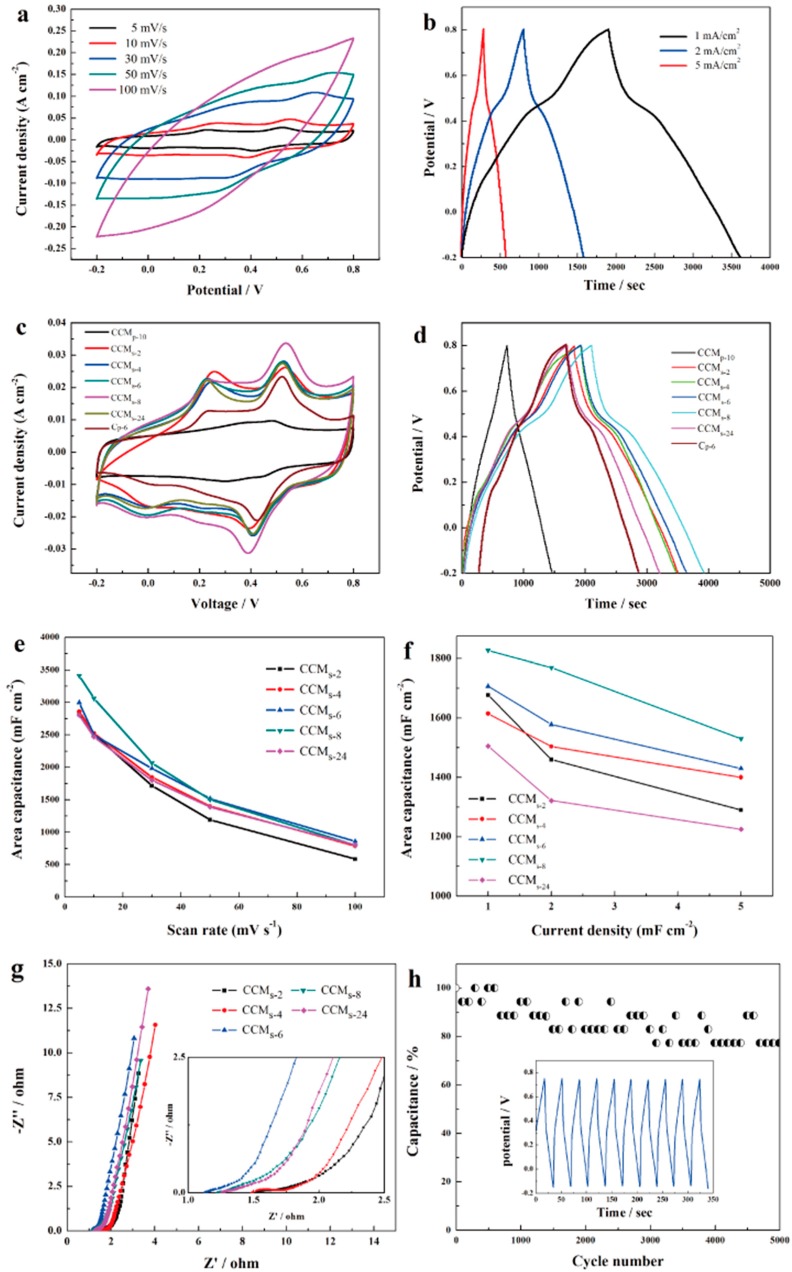
CV curve of CCM_s-6_ at different scan rates (**a**); GCD curves of CCM_s-6_ at different current densities (**b**); comparison of CV loops of the samples (**c**); comparison of GCD curves of the samples (**d**); rate capacity of CCM_s-2_, CCM_s-4_, CCM_s-6_, CCM_s-8_ and CCM_s-24_ (**e**,**f**); EIS spectrum of CCM_s-2_, CCM_s-4_, CCM_s-6_, CCM_s-8_ and CCM_s-24_ (**g**); and the capacitance retention of CCM_s-6_ after 5000 times of cycling (**h**).

In order to investigate the effect of polymerization time on PANI in CCM_p-10_, CHI660E was also used to assess the electrochemical performance of the samples. Figure 10a showed the cyclic voltammogram curves of the CCM_s-6_ electrode at different scan rates in 1 M H_2_SO_4_. Typical redox peaks of PANI could be clearly observed under low scan rates with oxidation peaks located at about 0.22 V and 0.52 V, and a reduction peak at 0.41 V demonstrated the great capacitance performance of CCM_s-6_. However, the redox peaks decreased with the increase of scan rate, and at a high scan rate of 100 mV/s, the cathodic peaks and the anodic peaks disappeared totally, which may due to the lack of time for redox reactions. Further, the shape of the CV loops of CCM_s-6_ expanded stably, which suggested the good reversibility and charge propagation of the electrodes. The capacitance of the CCM_s-6_ is calculated to be 3000, 2500, 1983, 1519, and 860 mF·cm^−2^ at 5, 10, 30, 50, and 100 mV·s^−1^, respectively. To demonstrate the effect of polymerization time on the electrochemical performance of the electrodes, comparisons of CV curves at 5 mV·s^−1^ of different samples were carried out. As shown in Figure 10c, CCM_s-2_, CCM_s-4_, CCM_s-6_, CCM_s-8_, CCM_s-24_, and C_p-6_ all possessed higher integral area than CCM_p-10_, which indicates that the deposited PANI indeed enhanced the capacitance performance of CCM_p-10_. However, PANI directly deposited on A-CFP (C_p-6_) show limited enhancement, which may due to that 6 h of polymerization could not fully utilize the A-CFP surface. The partial growth of PANI on the surface of A-CFP has been investigated in the SEM images before (Figure 3a). The specific capacitance of CCM_s-2_, CCM_s-4_, CCM_s-6_, CCM_s-8_, CCM_s-24_, and C_p-6_ was calculated to be 2810, 2862, 3082, 3412, 2803, and 2008 mF·cm^−2^. After then, galvanostatic charging/discharging measurements were further used to characterize the electrochemical performance of the samples. Figure 10b shows the GCD curves of CCM_s-6_ at different current densities verified from 1 to 5 mA·cm^−2^. The specific area capacitance was calculated to be 1706, 1560, and 1430 mF·cm^−2^ at corresponding current densities of 1, 2, and 5 mA·cm^−2^. The capacitance decreased by increasing the current densities, which were consistent with the CV measurement. Notably, this is a remarkable value when compared to the recently reported electrodes listed in Table 1. Comparison of GCD curves of CCM_p-10_, CCM_s-2_, CCM_s-4_, CCM_s-6_, CCM_s-8_, CCM_s-24_, and C_p-6_ were shown in Figure 10d. Compared to CCM_p-10_, all the discharge curves were not ideal straight lines, which demonstrated a faradic reaction process of PANI. The shape of CCM_s-6_ was almost symmetric, which suggested the ideal capacitive nature of the electrode. Figure 10e,f illustrated the rate capability of the samples. The practical capacitance of the samples increased with the increasing polymerization time, but due to the acceleration decrease of capacitance at high scan rates and current densities for CCM_s-8_, we chose CCM_s-6_ as electrodes to fabricate symmetrical supercapacitors. The electrochemical performance of the samples was further evaluated by electrical impedance specteoscopy (EIS), and the Nyquist plots are shown in Figure 10g. In the EIS spectrum, the ohmic internal resistance (Rs) is equal to the value of intercept on the X-axis at a high frequency, while the diameter of the semicircle represents the charge transfer resistance (Rct) [38]. The Rs for CCM_s-6_ was the lowest among the samples, and the value was about 1.1 Ω. Moreover, the low frequency line of all the plots showed a nearly vertical shape with a slope of about 60°, which suggested faster ion diffusion behavior. An obvious semicircle shape could be observed in CCM_s-4_, indicating relatively higher interfacial resistance and decreased conductivity. This may be explained the fact that the synthesis of PANI could be accomplished in 4 h and would suffer from hydrolysis in the following reaction time [39]. Further, the extra synthesized PANI may block the diffusion channel of ions into the inner space of the electrodes.

For the practical application, the cycle stability of CCM_s-6_ was tested by conducting continuous galvanostatic charging/discharging measurements at 50 mA·cm^−2^. The electrode retained approximately 76.5% of its initial capacitance (850 mF·cm^−2^) after 5000 times cycle and even retained 70.6% of the capacitance after 10,000 times of cycling. This result confirmed the good stability of the carbon fiber paper-based electrode.

**Figure 11:**
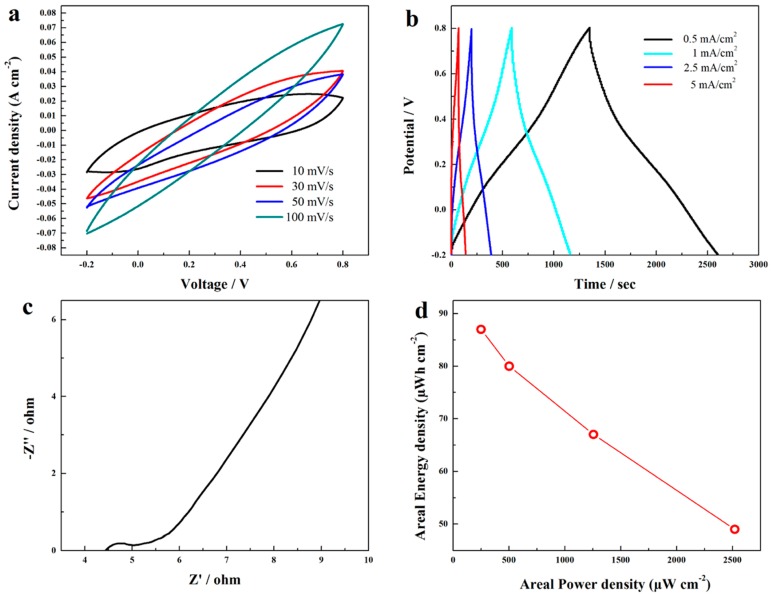
CV loops of the assembled symmetric supercapacitor at different scan rates (**a**); GCD curves of the ASSC at different current densities (**b**); EIS of the ASSC (**c**); and comparison of power density and energy density of the ASSC (**d**).

The practical electrochemical performance of the electrode was further evaluated by assessing the property of the assembled symmetric supercapacitors. Figure 11a showed the CV curves of the ASSC at different scan rates. Although the shape of the CV loops become narrower compared to single electrode, which may be caused by the imperfect contact between the two electrodes, the symmetric shape was still retained. For GCD curves of the ASSC (Figure 11b), all the curves show symmetric and straight lines, indicating the ideal capacitive nature of the sample, and a high area capacitance of 626 mF·cm^−2^ was achieved at 0.5 mA·cm^−2^. The EIS spectrum of the ASSC showed the resistance of the supercapacitor was rather small, and the straight line at low frequency with a slop of about 60° suggested the ideal capacitive nature (Figure 11c). Further, as shown in Figure 11d, the ASSC achieved a high power density of 250.3 μW cm^−2^ with energy density of 87 μWh·cm^−2^ at 0.5 mA·cm^−2^.

## 4. Conclusions

In summary, we have successfully fabricated A-CFP/CNFs-CNTs/PANI hybrid paper electrode for supercapacitors using a simple, low-cost, and effective method. The existence of the CNFs/CNTs mixture provided more active sites for the polymerization of PANI and thus achieved enhanced capacitance of 1706 mA·cm^−2^ and long-term cycle stability (it retained 70.6% of its initial capacitance). Further, the assembled symmetric supercapacitors delivered high area-capacitances of 1251 mF·cm^−2^, 87 μWh·cm^−2^ of area energy density and 250.3 μW·cm^−2^ of power density. It is believed that the as-prepared products could be promising electrodes for supercapacitors.

## Figures and Tables

**Table 1:** Comparison of the capacitance of different electrode materials.

Electrode Materials	Electrolyte	Capacitance (mF·cm^−2^)	Ref.
CNT/PANI hydrogel film	1 M H_2_SO_4_	680	[40]
Graphene/PANI aerogels	H_2_SO_4_/PVA	679	[41]
Carbon fiber paper	6 M KOH	750	[42]
Graphene/polypyrrole	H_2_SO_4_/PVA	477	[43]
CNT/polypyrrole	H_3_PO_4_/PVA	27.8	[44]
Organometal halide perovskite solar cells/CNT/PANI	PVA	422	[45]
